# Wear debris pseudotumor following total knee arthroplasty: a case report

**DOI:** 10.1186/1752-1947-3-9304

**Published:** 2009-11-29

**Authors:** Andreas F Mavrogenis, George N Nomikos, Vasileios I Sakellariou, George I Karaliotas, Panayiotis Kontovazenitis, Panayiotis J Papagelopoulos

**Affiliations:** 1 First Department of Orthopaedics, ATTIKON University General Hospital, Athens University Medical School, Athens, Greece

## Abstract

**Introduction:**

In patients who have undergone a total joint replacement, any mass occurring in or adjacent to the joint needs thorough investigation and a wear debris-induced cyst should be suspected.

**Case presentation:**

An 81-year-old man presented with a painful and enlarging mass at the popliteal fossa and calf of his right knee. He had had a total right knee replacement seven years previously. Plain radiographs showed narrowing of the medial compartment. Magnetic resonance imaging showed a cystic lesion at the postero-medial aspect of the knee joint mimicking popliteal cyst or soft tissue sarcoma. Fine needle aspiration was non-diagnostic. A core-needle biopsy showed metallosis. Intraoperative findings revealed massive metallosis related to extensive polyethylene wear, delamination and deformation. Revision knee and patella arthroplasty was carried out after a thorough debridement of the knee joint.

**Conclusion:**

Long-term follow-up is critical for patients with total joint replacement for early detection of occult polyethylene wear and prosthesis loosening. In these cases, revision arthroplasty may provide a satisfactory knee function.

## Introduction

Orthopaedic surgeons must consider several pathologic entities for patients presenting with painful, enlarging masses of the knee joint. Among these are primary soft tissue or bone sarcomas, metastatic carcinomas to bone and aneurysms of the popliteal artery, popliteal cysts infection, deep vein thrombosis, or cellulitis [1,2]. Differential diagnosis should also include wear debris and metallosis, and aseptic loosening of the knee prostheses especially among patients with prior total knee replacement.

Imaging studies and biopsy are necessary to evaluate the mass and to exclude primary and metastatic bone and soft tissue tumors in these patients. Metastatic carcinomas to bone are rarely located around the knee joint; popliteal cysts, cellulitis, deep vein thrombosis and aneurysms can be evaluated using ultrasonography [1-3].

We present the case of a patient with total knee arthroplasty presenting with a painful enlarging soft tissue mass at the popliteal fossa and calf associated with metallosis and extensive polyethylene wear mimicking soft tissue sarcoma.

## Case presentation

An 81-year-old man was admitted to our institution with a five-month history of a painful enlarging mass at the right popliteal fossa and calf. There was no history of trauma. His past medical history was unremarkable. He had undergone a primary total knee arthroplasty of his right knee seven years previously for degenerative knee osteoarthritis.

**Figure 1:**
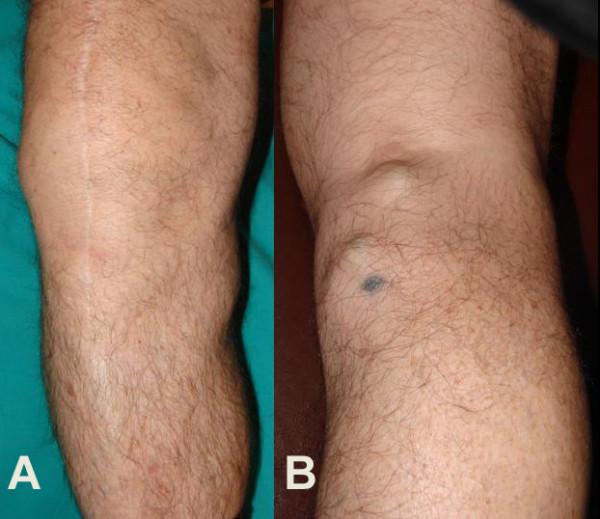
**(A, B) Photographs of the knee show a mass at the postero-medial aspect of the right knee, and two smaller darkish nodules in the popliteal fossa**.

Clinical examination showed painful restriction of the range of motion of the right knee, and a palpable mass at the posterior-medial aspect of the knee (Figure 1). The calf was slightly painful during palpation and passive stretching.

**Figure 2:**
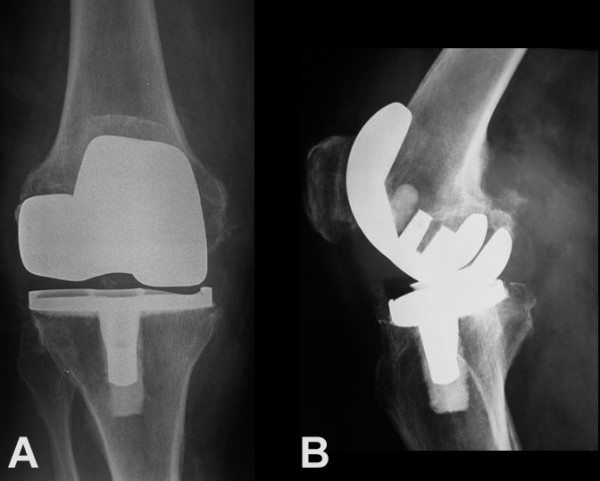
**(A) An anteroposterior radiograph of the right knee shows narrowing of the medial joint compartment, varus malalignment of the prosthetic joint, and radiolucent lines around femoral and tibial components**. **(B) **Lateral radiograph shows a soft tissue mass at the popliteal fossa.

**Figure 3:**
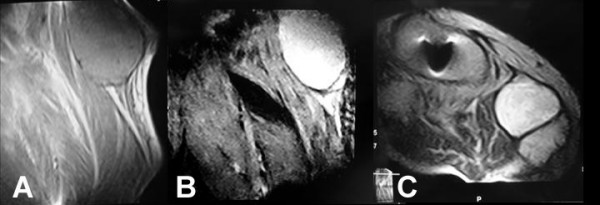
**(A) A sagittal T1-weighted magnetic resonance image of the right knee shows a low signal lesion with a maximal diameter of 8 cm at the origin of the medial head of the gastrocnemius muscle**. **(B) **A sagittal T2-weighted magnetic resonance image shows a high signal fluid-filled lesion with inner septums and thin outer wall. **(C) **A transverse T2-weighted magnetic resonance image shows a cystic fluid-filled lesion at the postero-medial aspect of the knee joint.

Plain radiographs of the right knee showed narrowing of the medial compartment, varus deformity of the arthroplasty, and features of loosening including radiolucent lines next to the femoral and tibial components. Some degree of polyethylene wear, especially in the medial compartment, was suspected (Figure 2). Magnetic resonance imaging of the right knee showed a cystic lesion located at the postero-medial aspect of the knee joint mimicking a popliteal cyst or soft tissue sarcoma (Figure 3). Plain radiographs of the chest were normal. A routine laboratory examination including complete blood cell count, serum chemistries, erythrocyte sedimentation rate and C-reactive protein reported values within normal ranges. Triplex ultrasonography showed no evidence of deep venous thrombosis.

Fine needle aspiration biopsy was carried out, but it was non-diagnostic. A core-needle biopsy revealed a black fluid containing thick, amorphous metallic debris and fragments of broken polyethylene. Histological sections showed necrobiotic tissue, wear debris and metallic particles, fibroblasts, osteoblasts, histiocytes and multinucleated giant cells. Nucleous atypia and mitotic features were not observed. Gram stain and cultures were negative for infection.

**Figure 4:**
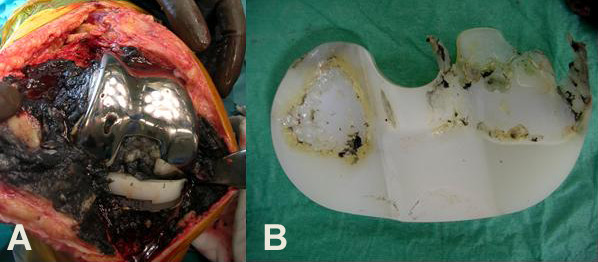
**Intraoperative photographs show (A) massive metallosis of the soft tissue adjacent to the prostheses, and (B) extensive wear, delamination and deformation of the polyethylene insert distributed asymmetrically over the medial and lateral joint surfaces**.

**Figure 5:**
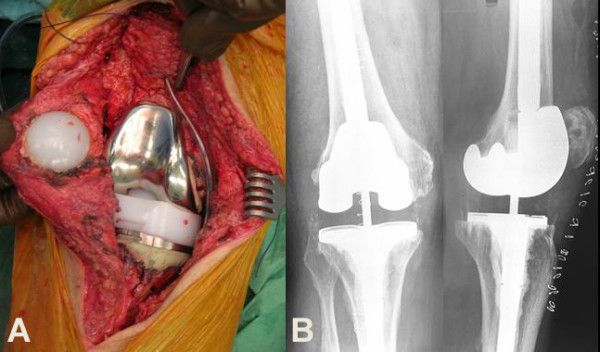
**(A) Intraoperative photograph after tricompartmental revision knee arthroplasty**. **(B) **Postoperative anteroposterior (left) and lateral (right) radiographs of the right knee.

Revision knee surgery showed massive metallosis, osteolysis of the distal femur and the proximal tibia and extensive polyethylene wear and deformation distributed asymmetrically over the medial and lateral joint surfaces (Figure 4). Debridement of the knee joint was done followed by revision of the femoral and tibial components and the polyethylene insert, in addition to patella arthroplasty (Figure 5). The patient's recovery was uncomplicated. His rehabilitation included strengthening and a range of motion exercises, which he started on the second postoperative day. The patient was discharged uneventfully after seven days of hospitalization. At the latest examination 2.5 years post-operatively the patient had a painless and stable knee joint without any clinical or imaging evidence of wear, metallosis or infection.

## Discussion

Previous studies have reported the formation of pelvic, thigh or calf masses related to osteolysis and wear debris in patients with hip and knee joint arthroplasties [1,2,4-11]. These masses have been reported up to eight years following primary total joint replacement [10]. In all cases, as in our case, the diagnosis was osteolysis and foreign body reaction caused by polyethylene wear and metallic debris accumulation.

Significant factors associated with implant wear are the activity and body weight of the patient, the type of the articulation (constrained or non-constrained), the design of the implant, the bearing surfaces, the alignment and dynamic balancing of the prosthetic joint, and the thickness, the quality and the oxidation methods of sterilization of the polyethylene insert [4,5,11,12]. The combination of these factors may result in the generation of significant quantities of particulate debris. Concomitant metallosis leads to a vicious cycle of failure with increasing abrasive wear of the articulation. In our patient, the right knee mass consisted of wear debris and metallic particles. Intraoperative findings showed massive metallosis in the soft tissues around the tibial prosthesis, and periprosthetic osteolysis secondary to extensive wear, delamination and deformation of the polyethylene insert.

Wear debris from implanted prostheses is generated by wear or abrasion, adhesion and fatigue [13]. *In vitro *studies have shown that macrophages exposed to wear particles produce cytokines that induce bone resorption [5,11]. Titanium particulate debris is usually found in the soft tissues adjacent to titanium prostheses, including the synovial layer and joint capsule [14]. Titanium debris can accumulate in the lymph nodes, liver and spleen, and lead to prosthetic loosening by inciting a foreign body reaction. Prosthetic loosening leads to increased micromotion at the component interface, which then generates further particulate debris [13].

The generation of excessive titanium debris can form radiodense masses around the joint. However, considering that titanium particulate debris has small size ranging from 1 μm to 20 μm, a large quantity of particles are required for radiographic evidence [13].

A painful mass emerged at the posterior-medial aspect of a patient's prosthetic knee joint seven years after a total knee replacement operation. The mass consisted of wear debris and metallic particles. Operative findings were massive metallosis in the soft tissues of the knee joint, periprosthetic osteolysis and extensive polyethylene wear, delamination and asymmetrical deformation. The polyethylene insert was the source of wear debris. The synovial fluid with wear particles drained underneath the fascia and the medial head of the gastrocnemius muscle in the popliteal fossa and the calf, and progressively enlarged with the accumulation of wear debris and metallic particles. A granulomatous tissue mass formed, mimicking a popliteal cyst or a sarcoma.

## Conclusion

In patients with total joint replacement, any mass occurring in or adjacent to the joint needs thorough investigation. A wear debris-induced cyst should be taken into account. Long-term follow-up is critical in patients with total joint replacement for early detection of occult polyethylene wear and prosthesis loosening. In these cases, revision arthroplasty is mandatory for good knee function.

## Consent

Written informed consent was obtained from the patient for publication of this case report and accompanying images. A copy of the written consent is available for review by the Editor-in-Chief of this journal.

## Competing interests

The authors declare that they have no competing interests.

## Authors' contributions

AFM, GNN, VIS, GIK, PK and PJP were the operating surgeons. All authors analyzed and interpreted the patient data regarding the diagnosis and management, studied the related literature and wrote the paper. All authors read and approved the final manuscript.
